# Underlying conditions associated with adverse COVID-19 treatment outcomes in selected Kenyan hospitals, October 2020 to December 2021

**DOI:** 10.1080/16549716.2025.2572010

**Published:** 2025-11-17

**Authors:** Dickens O. Onyango, Wanjiru Waruiru, Victor Omodi, Jonesmus Wambua, Peninah Masibo, Frank Victor Otieno, Wilfred Kazungu, Stella Njuguna, Christina Moore, Daniel Maangi, Jacques Muthusi, Anne Njoroge, Peninah Munyua, Anthony Waruru, Mary Mwangome, Emmanuel Okunga, George W. Rutherford

**Affiliations:** a Department of Health, County Government of Kisumu, Kisumu, Kenya; b Global Programs for Research and Training, University of California San Francisco, Nairobi, Kenya; c Department of Health, County Government of Nairobi, Nairobi, Kenya; d Department of Health, County Government of Kilifi, Kilifi, Kenya; e Division of Global HIV & TB (DGHT), Global Health Center, US Centres for Disease Control and Prevention, Nairobi, Kenya; f International Training & Education Center for Health (ITECH), University of Washington, Seattle, WA, USA; g Division of Disease Surveillance and Response, Ministry of Health, Nairobi, Kenya; h Institute for Global Health Sciences, University of California, San Francisco, USA

**Keywords:** hypertension, diabetes, noncommunicable diseases, HIV, COVID-19

## Abstract

**Background:**

Data are limited on the impact of chronic underlying conditions on COVID-19 treatment outcomes in sub-Saharan Africa.

**Objectives:**

Determine the effect of underlying conditions on COVID-19 severity and treatment outcomes in Kenya.

**Methods:**

We conducted a retrospective cohort study using routine medical records from Kenya’s three large COVID-19 treatment centers. We examined two outcomes: mortality and clinical severity. Patients with lower respiratory tract illness without tachypnoea or difficulty in breathing were considered to have mild COVID-19. Underlying conditions were based on documentation in medical records. Logistic regression models assessed associations between underlying conditions and COVID-19 severity and mortality.

**Results:**

Among the 1,123 hospitalized COVID-19 patients included in the analysis, 59% (*n* = 664) had at least one underlying condition, 24.9% (*n* = 261) had severe disease, and 32.5% (*n* = 365) died. Hypertension (61.1%; *n* = 409), diabetes (38.9%; *n* = 258), and HIV (14.2%; *n* = 94) were the most common underlying conditions. Deaths were significantly higher among hospitalized COVID-19 patients with underlying conditions (35.4%) than those without (28.3%) (*p* = 0.01). The adjusted odds of severe COVID-19 were 50% higher among patients with hypertension (adjusted odds ratio [aOR] 1.50; 95% confidence interval [CI] 1.11–2.02). Mortality during COVID-19 hospitalization was nearly threefold higher among patients with heart disease (aOR 2.82; 95% CI 1.18–6.74) and 37% higher among patients with diabetes (aOR 1.37; 95% CI 1.00–1.88).

**Conclusion:**

Patients with underlying conditions hospitalized for COVID-19 were more likely to die than those without. Hypertension was independently associated with disease severity, while heart disease and diabetes were independently associated with death during COVID-19 treatment.

## Paper context

- **Main findings:** More than half of patients hospitalized with COVID-19 had underlying conditions, and mortality was significantly higher among those with chronic heart disease and hypertension.
- **Added knowledge:** The study highlights the potential impact of non-communicable diseases and chronic infectious diseases, such as HIV, on infectious disease pandemics in low and middle income countries.
- **Global health impact for policy and action:** Strengthening integrated care for non-communicable and infectious diseases is critical to mitigate the impact of future pandemics in low- and middle-income countries undergoing epidemiological transition.

## Background

The severe acute respiratory syndrome type 2 (SARS CoV-2) pandemic and its clinical manifestation (coronavirus disease [COVID-19]) devastated global public health, with more than 762 million confirmed cases and over 6.8 million deaths reported globally as of July 2023 [1]. By the same time, in Kenya, the number of confirmed COVID-19 cases exceeded 340,000, with over 5,600 deaths reported [1]. Underlying conditions are long-term medical conditions in individuals presenting with an illness, such as noncommunicable diseases (NCD) and chronic communicable diseases, including HIV infection [2]. Many studies, particularly from high-income countries, associated NCDs (cardiovascular diseases, diabetes, obesity, asthma, and chronic renal disease) with severe symptoms of COVID-19 or death [3–9]. In Kenya, studies conducted during the first year of the pandemic found that COVID-19 patients with underlying conditions were over twice as likely to die during treatment compared to those without underlying conditions [10,11]. However, these studies did not include data from COVID-19 driven by newer variants, especially Delta, and did not examine the effects of comorbid conditions on disease severity and mortality. Data on the most common underlying conditions in sub-Saharan Africa (SSA), the role of chronic communicable diseases, especially HIV, and the distribution of underlying conditions by patient characteristics and across the successive waves of the pandemic have primarily been from Southern Africa [12–14]. However, there is a lack of data from Eastern Africa, leading to a limited understanding of the implications of these underlying conditions on COVID-19 treatment outcomes in the region. A better understanding of the impact of NCDs and chronic communicable diseases on COVID-19 outcomes is crucial for the care of COVID-19 patients and preparing for and responding to future infectious disease pandemics [15].

COVID-19 struck as NCDs, such as cardiovascular disease, diabetes, obesity, asthma, and chronic renal diseases), were recognized to be rising globally, with 22% of the world’s population estimated to have had at least one prevalent chronic disease; one-third of these had at least two long-term conditions [5,9,16,17]. NCDs are a particular concern in SSA, where their prevalence is rising faster than in the rest of the world [18,19]. In SSA, NCDs account for nearly half of all hospital admissions [18,19]; the increasing NCD burden in SSA has been attributed to changing lifestyles and increasing life expectancy [20,21]. NCDs are now believed to be more common among people living with HIV (PLHIV) than HIV-uninfected people due to chronic inflammation from HIV infection, the side effects of antiretroviral therapy (ART), and increased longevity [20,21]. NCDs are becoming a major public health concern in SSA countries with a high HIV burden [22]. In Kenya, for instance, over half of the population are thought to have at least one prevalent NCD, with a higher prevalence among PLHIV [23]. In the 2015 Stepwise Survey for Noncommunicable Disease Risk Factors, obesity (27.9%), hypertension (23.8%), and type 2 diabetes (3.1%), were the most common risk factors in the country [24].

Early in the COVID-19 pandemic, it was feared that PLHIV would be predisposed to severe or fatal COVID-19 due to immunosuppression or comorbidity with NCDs. To date, there are conflicting reports about how HIV affected COVID-19 outcomes in high-HIV prevalence countries. While two studies conducted in South Africa [13,25] and a WHO analysis of over 190,000 patients [26] found an association between HIV and severity and mortality from COVID-19, a meta-analysis of 16 studies found no significant association between HIV and mortality from COVID-19 [27]. In this study, we examined the distribution of underlying conditions, including NCDs and HIV infection, among people hospitalized with COVID-19 using routine data from three high HIV prevalence counties in Kenya (Nairobi, Kisumu, and Kilifi). We further explored how these underlying conditions were associated with COVID-19 severity and treatment outcomes.

## Methods

### Study design and setting

This retrospective cohort study abstracted data from the routine medical records of patients admitted with COVID-19 in three hospitals in Kenya: Mbagathi County Referral Hospital in Nairobi County, Jaramogi Oginga Odinga Teaching and Referral Hospital in Kisumu County, and Kilifi County Referral Hospital in Kilifi County. Data was obtained between 5 October 2022 to 24 February 2023. The three hospitals were purposively selected based on the high number of COVID-19 cases reported during the selected study period and the relatively high prevalence of HIV in the three counties. Nairobi is a gateway for international travel and had both the first confirmed COVID-19 patient and the highest COVID-19 burden [11,28]. The more rural Kilifi County had a high COVID-19 burden early in the pandemic, probably due to tourism activities and its proximity to the port city of Mombasa. Kisumu County, which borders Uganda through Lake Victoria, was the epicenter of the Delta wave in the second year of the pandemic. By mid-2021, COVID-19 seroprevalences in Kisumu, Nairobi, and Kilifi counties were 42%, 52%, and 25%, respectively [28]. These three referral facilities served as COVID-19 isolation and treatment centers for their respective counties. HIV prevalence among people aged 15–64 years in these counties ranged from 17.5% in Kisumu, 3.8% in Nairobi and 2.3% in Kilifi in a 2018 population-based survey [29]. These three counties were also COVID-19 hotspots during various waves [11]. Kenya had experienced five COVID-19 waves by December 2021, each driven by a different variant. The first wave lasted from March to September 2020, the second (Alpha variant) from October to January 2021, the third (Alpha and Beta variants) from February to May 2021, the fourth (Delta variant) from June to November 2021, and the fifth began in December 2021 (Omicron variant) [30].

### Study population

We abstracted data from medical records for patients hospitalized for COVID-19 in the participating health facilities between 1 October 2020, and 31 December 2021. We included patients with either a positive COVID-19 rapid test, polymerase chain reaction, or presumptively treated for COVID-19.

### Data collection and management

Data were collected from 5 October 2002 to 24 February 2023. Trained research assistants abstracted data from the COVID-19 treatment records onto tablets using a structured form implemented using Open Data Kit (ODK). The extracted variables included socio-demographic characteristics (age in years, sex, and date of hospitalization), clinical characteristics, including underlying conditions, HIV status, and outcome of treatment (death, discharge, or referral). Although we attempted to abstract the COVID-19 vaccination status, this information was unavailable for over 90% of patients and was therefore excluded from the analysis.

We considered participants to have underlying conditions if there was documentation in their medical records that they were also on treatment for another condition. We evaluated the presence of the most prevalent underlying conditions, including hypertension, cancer, diabetes, HIV, chronic kidney disease, chronic heart disease, chronic respiratory diseases, liver disease, and obesity. We created a binary variable for the presence of underlying conditions. A value of ‘Yes’ was assigned if a patient had at least one underlying condition, and ‘No’ if otherwise. These underlying conditions were classified following a hierarchical structure to ensure that patients with multiple underlying conditions were counted only once. We defined COVID-19 severity according to the Ministry of Health’s COVID-19 management guidelines, which classified patients with upper respiratory tract symptoms without dehydration, sepsis, or shortness of breath as uncomplicated COVID-19 and patients with signs of lower respiratory tract infection without difficulty in breathing or tachypnea as mild COVID-19 [31]. We classified participants as having severe COVID-19 if they required supplemental oxygen, had a respiratory rate >30 breaths per minute or an SpO_2_ < 93% in adolescents or adults or <90% in children aged <15 years, required mechanical ventilation, had shock, or required intensive care, at any point during hospitalization. We categorized COVID-19 treatment outcomes as alive or dead from any cause during admission (based on documentation in the medical records) for COVID-19 and considered participants who were referred to other facilities as having survived COVID-19.

### Data analysis

We used descriptive statistics to summarize the characteristics of the study population, including age, sex, underlying conditions, and outcomes. Our primary outcomes were severe COVID-19 and treatment outcome as either alive or dead. We used chi-square tests to compare the distribution of underlying conditions by the two study outcomes: severity of COVID-19 cases and treatment outcome (alive versus dead) and mixed-effects logistic regression models to account for clustering at facility level and assess the independence of association between underlying conditions and COVID-19 severity and mortality. Factors with a p-value ≤0.2 in bivariate analysis were subjected to multivariate analysis. In the multivariate analysis, we adjusted for factors that showed significance in the bivariate analysis. The selection of factors to be assessed during regression was based on existing literature and clinical understanding. During analysis, p-values < 0.05 were considered statistically significant. We used R 4.2.3 (R Project for Statistical Computing, Vienna, Austria) to perform the analysis.

### Ethical considerations

This study was conducted in accordance with the Declaration of Helsinki and was reviewed and approved by AMREF Health Africa Ethics and Scientific Review Committee (approval number P1233/2022), the University of California, San Francisco Human Research Protection Program Institutional Review Board (IRB approval number 22–37353), the US Centers for Disease Control and Prevention (CDC) (approval number CGH-KEN-7/26/22–90353) and the institutional ethics review committees from the three participating hospitals. **Due to the study’s retrospective nature and the use of de-identified data, the requirement for individual patient consent was waived**. The Kenya National Commission for Science, Technology and Innovation (NACOSTI) granted the permit to conduct this study (License No: NACOSTI/P/22/19903).

## Results

A total of 1,123 patients hospitalized with COVID-19 were included in this analysis. Of these, 59.1% (*n* = 664) had one or more underlying conditions, 53.0% (*n* = 595) were male, and 39.4% (*n* = 443) were aged 60 years or older (Table 1). Nearly half (48.4%; *n* = 543) of the patients included in this study were from Kisumu County, followed by 38.5% (*n* = 432) in Nairobi County and 13.2% (*n* = 148) in Kilifi County. Of all the patients hospitalized for COVID-19, 93.2% (*n* = 1047) had information that could be used to categorize disease severity. Among these patients, 24.9% (*n* = 261) had severe disease. Severe COVID-19 was not significantly more common among patients with underlying conditions (57.9%; *n* = 151) than patients without underlying conditions (42.1%; *n* = 110) (p-value = 0.36). While 64.6% (*n* = 725) of the hospitalised patients were discharged alive, 32.5% (*n* = 365) died during their hospitalization.

**Table 1.** Sociodemographic characteristics of patients hospitalized with COVID-19 in selected hospitals stratified by the presence of underlying conditions, Kenya, October 2020 – December 2021.

	Had underlying condition	No underlying condition		Overall
Characteristic	(*N* = 664) n (%)	(*N* = 459) n (%)	P-value^¢^	(*N* = 1123) n (%)
Age Category in years* (*N* = 1120)				
0 – 19	7 (24.1)	22 (75.9)	<0.001	29 (2.6)
20 – 29	43 (50.0)	43 (50.0)		86 (7.7)
20 – 39	72 (45.3)	87 (54.7)		159 (14.2)
40 – 49	97 (55.1)	79 (44.9)		176 (15.7)
50 – 59	151 (66.5)	76 (33.5)		227 (20.2)
60+	293 (66.1)	150 (33.9)		443 (39.4)
Sex** (*N* = 1119)				
Female	333 (63.5)	191 (36.5)	0.004	524 (46.7)
Male	328 (55.1)	267 (44.9)		595 (53.0)
County (*N* = 1123)				
Kilifi	104 (70.3)	44 (29.7)	0.001	148 (13.2)
Kisumu	330 (60.8)	213 (39.2)		543 (48.4)
Nairobi	230 (53.2)	202 (46.8)		432 (38.5)
COVID-19 Severity*** (*N* = 1047)				
Severe	151 (57.9)	110 (42.1)	0.358	261(24.9)
Mild/Moderate	480 (61.1)	306 (38.9)		786(75.1)
COVID-19 wave**** (*N* = 1119)				
2(Oct20-Dec20)	70 (54.7)	58 (45.3)	0.690	128 (11.4)
3(Feb21-May21)	105 (60.0)	70 (40.0)		175 (15.6)
4(Jun21-Oct21)	436 (59.9)	292 (40.1)		728 (65.1)
5(Nov21-Dec22)	50 (56.8)	38 (43.2)		88 (7.9)
Treatment outcome (*N* = 1123)				
Dead	235 (64.4)	130 (35.6)	0.032	365 (32.5)
Discharged	408 (56.3)	317 (43.7)		725 (64.6)
Referral	21 (63.6)	12 (36.4)		33 (2.9)

Of all (*N* = 1,123) patients hospitalized for COVID-19, 35.9% (*n* = 403) had one underlying condition, while 23.2% (*n* = 261) had multiple (two or more) underlying conditions (Table 2). The proportion of COVID-19 patients with multiple underlying conditions was highest among patients aged 50–59 years (30.4%; *n* = 69) and those aged 60 years or more (28.9%; *n* = 128). Among patients with underlying conditions, the frequency of multiple underlying conditions was slightly higher in females (40.8%; *n* = 136) than in males (37.2%; *n* = 122). The top three underlying conditions were hypertension (61.6%, *n* = 409), diabetes (38.9%, *n* = 258), and HIV (14.2%, *n* = 94). Patients aged 60 years and above had the highest prevalence of hypertension (78.8%; *n* = 231), while patients aged 50–59 years had the highest prevalence of diabetes (49.7%, *n* = 75). Only 9.3% (*n* = 105) of patients hospitalized with COVID-19 had their HIV status documented in their medical records, of whom 89.5% (*n* = 94) were HIV-infected. ART status was available for 73.4% (*n* = 69) with a documented HIV-positive status, 75.0% (*n* = 52) of these were on a dolutegravir (DTG)-based regimen.

**Table 2.** Distribution of underlying conditions among patients hospitalized with COVID-19 in selected hospitals by age and sex, Kenya, October 2020 – December 2021.

		Age Group in Years [N = 1120*] (Col %)				Sex [N = 1119**] (Col %)	
	All	<40	40–49	50–59	60+	Male	Female
Characteristic	N = 1123%)	N = 274(%)	N = 176(%)	N = 227(%)	N = 443(%)	N = 595(%)	N = 524(%)
Underlying conditions							
No	459 (40.9)	152 (55.5)	79 (44.9)	76 (33.5)	150 (33.9)	267 (44.9)	191 (36.5)
Yes	664 (59.1)	122 (44.5)	97 (55.1)	151 (66.5)	293 (66.1)	328 (55.1)	333 (63.5)
Number of Underlying conditions							
1	403 (60.7)	98 (80.3)	57 (58.8)	82 (54.3)	165 (56.3)	206 (62.8)	197 (59.2)
2+	261 (39.3)	24 (19.7)	40 (41.2.)	69 (45.7)	128 (43.7)	122 (37.2)	136 (40.8)
Hypertension							
No	255 (38.4)	99 (81.1)	41 (42.3)	52 (34.4)	62 (21.2)	129 (39.3)	126 (37.8)
Yes	409 (61.6)	23 (18.9)	56 (57.7)	99 (65.6)	231 (78.8)	199 (60.7)	207 (62.2)
Diabetes							
No	406 (61.1)	98 (80.3)	62 (63.9)	76 (50.3)	170 (58.0)	196 (59.8)	209 (62.8)
Yes	258 (38.9)	24 (19.7)	35 (36.1)	75 (49.7)	123 (42.0)	132 (40.2)	124 (37.2)
Cancer							
No	658 (99.1)	122 (100.0)	97 (100.0)	147 (97.4)	291 (99.5)	291 (99.3)	331 (99.4)
Yes	6 (0.9)	0 (0.0)	0 (0.0)	4 (2.6)	2 (0.5)	4 (0.7)	2 (0.6)
HIV							
Negative	11 (1.7)	4 (3.3)	2 (2.1)	1 (0.7)	4 (1.4)	6 (1.8)	5 (1.5)
Positive	94 (14.2)	31 (25.4)	23 (23.7)	25 (16.6)	15 (5.1)	40 (12.2)	54 (16.2)
Unknown	559 (84.2)	87 (71.3)	72 (74.2)	125 (82.8)	274 (93.5)	282 (86.0)	274 (82.3)
Kidney disease							
No	625 (94.1)	116 (95.1)	94 (96.9)	145 (96.0)	269 (91.8)	308 (93.9)	314 (94.3)
Yes	39 (5.9)	6 (4.9)	3 (3.1)	6 (4.0)	24 (8.2)	20 (6.1)	19 (5.7)
Liver diseases							
No	661 (99.5)	122 (100.0)	96 (99.0)	151 (100.0)	291 (99.3)	327 (99.7)	331 (99.4)
Yes	3 (0.5)	0 (0.0)	1 (1.0)	0 (0.0)	2 (0.7)	1 (0.3)	2 (0.6)
Respiratory diseases							
No	594 (89.5)	102 (83.6)	82 (84.5)	140 (92.7)	269 (91.8)	289 (88.1)	302 (90.7)
Yes	70 (10.5)	20 (16.4)	15 (15.5)	11 (7.3)	24 (8.2)	39 (11.9)	31 (9.3)
Heart diseases							
No	642 (96.7)	120 (98.4)	96 (99.0)	148 (98.0)	277 (94.5)	312 (95.1)	327 (98.2)
Yes	22 (3.3)	2 (1.6)	1 (1.0)	3 (2.0)	16 (5.5)	16 (4.9)	6 (1.8)
Obesity							
No	6498 (97.7)	119 (97.5)	94 (96.9)	149 (98.7)	286 (97.6)	322 (98.2)	324 (97.3)
Yes	15 (2.3)	3 (2.5)	3 (3.1)	2 (1.3)	7 (2.4)	6 (1.8)	9 (2.7)

The third wave, which occurred from February to May 2021 (15.8%, *n* = 105) and fourth wave (65.7%, *n* = 436), which occurred from June to November 2021, had the highest proportions of hospitalized COVID-19 patients with underlying conditions. The proportion of hospitalized COVID-19 patients with multiple underlying conditions was highest during the fourth wave (61.3%, *n* = 160) (Table S1).

COVID-19 severity was significantly more common among participants with hypertension (79.7%; *n* = 314) than those without hypertension (72.3%; *n* = 472) (0.007) (Table 3) Mortality was significantly higher among patients with underlying conditions (35.4%; *n* = 235) than those without (28.3%; *n* = 130) (p-value = 0.01). Mortality was significantly higher among patients who had hypertension, diabetes, and heart disease than those who did not have these conditions. While mortality was higher among patients with chronic kidney disease than those without, the difference was borderline significant (0.06). Information on whether these conditions were controlled by medication was not available.

**Table 3.** Distribution of underlying conditions among patients hospitalized for COVID-19 in selected hospitals by the severity of COVID-19 and outcome of treatment, Kenya, October 2020 – December 2021.

Variable Name	Disease severity			Treatment outcome		
	Non-Severe, N = 261 n (%)	Severe, N = 786 n (%)	P-Value*	Alive, N = 758 n (%)	Dead, N = 365 n (%)	P-Value*
Had underlying condition			0.358			0.013
No	110 (26.4)	306 (73.6)		329 (71.7)	130 (28.3)	
Yes	151 (23.9)	480 (76.1)		429 (64.6)	235 (35.4)	
No. of underlying conditions			0.236			<0.006
None	110 (26.4)	306 (73.6)		329 (71.7)	130 (28.3)	
1	99 (25.9)	283 (74.1)		272 (67.5)	131 (32.5)	
2+	52 (20.9)	197 (79.1)		157 (60.2)	104 (39.8)	
Hypertension			0.007			0.005
No	181 (27.7)	472 (72.3)		503 (70.4)	211 (29.6)	
Yes	80 (20.3)	314 (79.7)		255 (62.3)	154 (37.7)	
Diabetes			0.305			0.009
No	206 (25.7)	596 (74.3)		601 (69.5)	264 (30.5)	
Yes	55 (22.4)	190 (77.6)		157 (60.9)	101 (39.1)	
Cancer			>0.999			0.670
No	260 (25.0)	782 (75.0)		753 (67.4)	364 (32.6)	
Yes	1 (20.0)	4 (80.0)		5 (83.3)	1 (16.7)	
HIV status			>0.999			0.329
HIV-negative	4 (36.4)	7 (63.6)		9 (81.8)	2 (18.2)	
HIV-infected	31 (35.6)	56 (64.4)		61 (64.9)	33 (35.1)	
Kidney_disease			0.785			0.06
No	252 (25.0)	756 (75.0)		737 (68.0)	347 (32.0)	
Yes	9 (23.1)	30 (76.9)		21 (53.8)	18 (46.2)	
Liver_disease			0.437			>0.9
No	260 (24.9)	785 (75.1)		756 (67.5)	364 (32.5)	
Yes	1 (50.0)	1 (50.0)		2 (66.7)	1 (33.3)	
Respiratory_diseases			0.430			0.2
No	247 (25.2)	733 (74.8)		716 (68.0)	337 (32.0)	
Yes	14 (20.9)	53 (79.1)		42 (60.0)	28 (40.0)	
Heart_diseases			0.189			0.007
No	259 (25.2)	768 (74.8)		749 (68.0)	352 (32.0)	
Yes	2 (10.0)	18 (90.0)		9 (40.9)	13 (59.1)	
Obesity			>0.999			0.8
No	258 (25.0)	774 (75.0)		747 (67.4)	361 (32.6)	
Yes	3 (20.0)	12 (80.0)		11 (73.3)	4 (26.7)	

In bivariate analysis, the odds of severe disease were 1.5 times higher among patients hospitalized for COVID-19 who had hypertension (odds ratio [OR] = 1.51; 95% CI 1.12–2.04) than patients without hypertension (Table 4). However, the odds of severe disease among known PLHIV (OR = 1.03; 95% CI 0.25–3.70) was similar to that of patients whose HIV status was negative. The odds of death among hospitalized COVID-19 patients were 44% higher among patients with hypertension (OR = 1.44; 95% CI 1.11–1.86) than those without hypertension, 46% higher among patients with diabetes than those without diabetes (OR = 1.46; 95% CI 1.10–1.95), and more than three-fold higher among patients with chronic heart disease than those without (OR = 3.07; 95% CI 1.31–7.51).

**Table 4.** Mixed effect logistic regression analysis of factors (underlying conditions) associated with severe disease and mortality among hospitalized COVID-19 patients in Kenya.

Variable Name		Disease Severity				Mortality			
		UOR (95% Cl)	p-value	AOR (95% CI)	p-value	UOR (95% Cl)	p-value	AOR (95% CI)	p-value
Hypertension	No	–	0.007	–	0.009	–	0.005	–	0.155
	Yes	1.51 (1.12–2.04)		1.50 (1.11–2.02)		1.44 (1.11–1.86)		1.23 (0.93–1.62)	
Diabetes	No	–	0.306	–	–	–	0.010	–	0.048
	Yes	1.19 (0.86–1.69)		–		1.46 (1.10–1.95)		1.37 (1.00–1.88)	
Heart diseases	No	–	0.138	–	0.173	–	0.010	–	0.020
	Yes	3.04 (0.87–19.18)		2.78 (0.64–12.13)		3.07 (1.31–7.51)		2.82 (1.18–6.74)	
Respiratory diseases	No	–	0.431	–	0.318	–	0.168	–	0.105
	Yes	1.28 (0.71–2.43)		1.36 (0.74–2.51)		1.42 (0.86–2.31)		1.52 (0.92–2.51)	
HIV Status	Negative	–	0.962	–	–	–	0.273	–	–
	Positive	1.03 (0.25–3.70)		–		2.43 (0.58–16.59)		–	
Kidney disease	No	–	0.785	–	–	–	0.068	–	–
	Yes	1.11 (0.54–2.51)		–		1.82 (0.95–3.46)		–	
Obesity	No	–	0.658	–	–	–	0.628	–	
	Yes	1.33 (0.42–5.89)		–		0.75 (0.21–2.22)		–	

On multivariate analysis, the adjusted odds of severe COVID-19 was 50% higher among people with hypertension than among those without (adjusted odds ratio [aOR] = 1.50; 95% CI 1.11–2.02). The adjusted odds of death during COVID-19 hospitalization was nearly threefold higher among patients with chronic heart disease than those without (aOR = 2.82; 95% CI 1.18–6.74) and 37% higher among people with diabetes than among those without (aOR = 1.37; 1.00–1.88).

## Discussion

In this retrospective cohort analysis, over half of patients admitted with COVID-19 had at least one underlying condition. Mortality during COVID-19 treatment was significantly higher among patients with underlying conditions, particularly chronic heart disease. Although the severity of COVID-19 was not significantly higher among those with underlying conditions, it was significantly higher among patients with hypertension than those without hypertension. The limited available data suggests that HIV infection did not contribute substantially to severe COVID-19 outcomes. Hypertension, diabetes, and HIV were the most common underlying conditions among patients hospitalized with COVID-19, as observed in other Kenyan studies [10,11]. The high burden of underlying conditions, particularly NCDs, observed in the study could be due to the epidemiological transition. Kenya, like many developing countries, is thought to be undergoing an epidemiologic transition, which involves a shift from a predominance of infectious diseases to a predominance of NCDs, due to improvements in sanitation, access to medical care and nutrition [32]. Among PLHIV, NCDs are disproportionately present and introduce a dual disease burden due to increased life expectancy among PLHIV, the inflammatory response to HIV, and the effects of antiretrovial therapy [20,21,23]. It is in this context, marked by a high burden of infectious diseases like HIV, fragile health systems with limited emergency and critical care capacity [33], and a rapidly growing prevalence of NCDs, the emergence and spread of infectious disease epidemics remains a grave threat.

Of note, over a fifth of patients admitted with COVID-19 had multiple underlying conditions. This is consistent with previous studies, which have reported an increase in the burden of multiple underlying conditions with increasing life expectancy and epidemiological transition [17,34]. In our study, multiple underlying conditions were associated with an increased risk of death, in line with earlier studies [35–37]. The number of underlying conditions has tended to increase with increase in age [17]. Thus, countries experiencing demographic transition are expected to have an increasing burden of individuals with multiple underlying conditions due in part to the increase in life expectancy. Additional focus on preventing and treating NCDs in low- and middle-income countries undergoing epidemiological transition could not only reduce morbidity and mortality from NCDs but also help in mitigating the impact of COVID-19. Furthermore, these findings suggest prioritization of targeted interventions that support patients who have multiple underlying conditions in addition to COVID-19.

In our analysis, less than one-tenth of admitted COVID-19 patients had their HIV status documented in their medical records. This reflects that HIV-related medical records are often managed separately from other medical records; hence, they may not be available for inpatient care. In a study that abstracted HIV status information from the medical records of inpatients in western Kenya, only 34% had their HIV status reported [38]. Alternatively, the poor availability of HIV status in medical records may be because some patients feel stigmatized about their condition and may not feel comfortable disclosing it to their health providers, family, or friends [39]. While the suboptimal availability of HIV status in medical records could imply low awareness of HIV status, this contradicts national estimates that indicate that nearly 90% of the Kenya population have been tested and are aware of their status [40]. The unavailability of HIV status among inpatients is a major concern, regardless of the reason. It limits the ability of attending clinicians to provide holistic care, prevents patients from taking ART, and predisposes patients to poor outcomes. Integrated health information systems that allow providers to access all medical records, especially HIV status and treatment history, may improve treatment decision-making. In this study, HIV was not significantly associated with severe COVID-19 or mortality. This contrasts with studies from South Africa [13,25] and the WHO study, which reported that HIV was associated with severe COVID-19 and mortality [26]. Our results align with a meta-analysis of 16 studies that also found nonassociation between HIV infection and fatal COVID-19 [27]. PLHIV receiving longitudinal follow-up in comprehensive care centers had better opportunities for timely diagnosis and referral if they are diagnosed with COVID-19. Additionally, most patients in the South African studies were on a tenofovir-based regimen [13], while three-quarters of our patients were on the more efficacious DTG-based regimen. These findings may suggest that HIV infection, particularly if well controlled, may not have contributed substantially to the severity and mortality of COVID-19 in these areas of Kenya. PLHIV who are on optimized ART regimens may achieve virological control and gain immune function, hence would be less predisposed to other infectious diseases.

## Limitations

This study relied on routine treatment records and did not verify NCD status through independent laboratory testing and clinical verification through measurements, such as blood pressure. This approach might have led to underestimating the true prevalence of NCDs and odds ratios. Additionally, the routine dataset we used had a large amount of missing data for some variables, especially HIV status, which had over 90% missing values. This poor documentation of HIV status may have contributed to underestimating the HIV burden in this study. Risk factors for death could not be evaluated using Cox proportional hazards regression because over half of the deceased did not have their dates of death recorded. Secondly, the distribution of underlying conditions in this study could have been influenced by selection bias. As awareness of the association of underlying conditions with severe or fatal COVID-19 increased over the pandemic period, patients with underlying conditions were preferentially hospitalized even if they had mild disease based on Ministry of Health recommendations. This could have led to an overestimation of the frequency of underlying conditions among hospitalized COVID-19 patients. Additionally, this could have confounded the association between the presence of underlying conditions and COVID-19 severity. Moreover, information on whether underlying conditions were controlled by medication or not was not available. Therefore, the impact of controlling the underlying conditions on COVID-19 severity and mortality could not be explored. This study included patients in whom SARS-CoV-2 infection was diagnosed using different testing methods. Limited access to PCR testing led to the inclusion of cases diagnosed by alternative modalities, including rapid antigen tests, potentially affecting diagnostic accuracy. However, the impact of this is minimal since only WHO approved tests were used. This study did not include patients without COVID-19 and therefore could not assess whether the distribution of underlying conditions in our study was different from the general population.

## Conclusions

In this study, more than half of the patients who were hospitalized with COVID-19 had underlying conditions. Mortality during COVID-19 treatment was significantly higher among patients with underlying conditions, particularly those with chronic heart disease. Disease severity was also significantly higher among patients with hypertension. Insufficient data limited our evaluation of the impact of HIV on COVID-19 severity and mortality; however, the limited available data suggest that HIV infection did not contribute substantially to severe COVID-19 outcomes, possibly due to effective ART and better health care seeking. Understanding the association between underlying conditions and COVID-19 outcomes is essential for guiding public health policies and interventions during future infectious disease epidemics. Efforts to prevent and control NCDs, particularly in the context of HIV, could be prioritized to mitigate the impact of multi-morbidity on COVID-19 outcomes and improve overall public health.

## Supplementary Material

Supplementary _table _clean copy.docx

STROBE_underlying conditions.docx

## Data Availability

The data that support the findings of this study are available from the corresponding author upon reasonable request.

## References

[1] World Health Organization. Who coronavirus (COVID-19) dashboard. 2023. Available from: https://covid19.who.int/

[2] Porta M, editor. Dictionary of epidemiology. 6th ed. Oxford: Oxford University Press; 2016.

[3] Barek MA, Aziz MA, Islam MS. Impact of age, sex, comorbidities and clinical symptoms on the severity of COVID-19 cases: a meta-analysis with 55 studies and 10,014 cases. Heliyon. 2020;6:e05684. doi: 10.1016/j.heliyon.2020.e05684 33344791 PMC7737518

[4] Phelps M, Christensen DM, Gerds T, et al. Cardiovascular comorbidities as predictors for severe COVID-19 infection or death. Eur Heart J Qual Care Clin Outcomes. 2021;7:172–10. doi: 10.1093/ehjqcco/qcaa081 33107909 PMC7665490

[5] Clark A, Jit M, Warren-Gash C, et al. Global, regional, and national estimates of the population at increased risk of severe COVID-19 due to underlying health conditions in 2020: a modelling study. Lancet Glob Health. 2020;8:e1003–e17. doi: 10.1016/S2214-109X(20)30264-3 32553130 PMC7295519

[6] Adab P, Haroon S, O’Hara ME, et al. Comorbidities and COVID-19. BMJ. 2022;377:o1431. doi: 10.1136/BMJ.o1431 35705219

[7] Puri A, He L, Giri M, et al. Comparison of comorbidities among severe and non-severe COVID-19 patients in Asian versus non-Asian populations: a systematic review and meta-analysis. Nurs Open. 2022;9:733–751. doi: 10.1002/nop2.1096 34761532 PMC8661719

[8] Fang X, Li S, Yu H, et al. Epidemiological, comorbidity factors with severity and prognosis of COVID-19: a systematic review and meta-analysis. Aging (Albany NY). 2020;12:12493–12503. doi: 10.18632/aging.103579 32658868 PMC7377860

[9] Zhang JJ, Dong X, Liu GH, et al. Risk and protective factors for COVID-19 morbidity, severity, and mortality. Clin Rev Allergy Immunol. 2023;64:90–107. doi: 10.1007/s12016-021-08886-7 35044620 PMC8767775

[10] Ombajo LA, Mutono N, Sudi P, et al. Epidemiological and clinical characteristics of patients hospitalised with COVID-19 in Kenya: a multicentre cohort study. BMJ Open. 2022;12:e049949. doi: 10.1136/bmjopen-2021-049949 PMC9121111 35589368

[11] Ngere P, Onsongo J, Langat D, et al. Characterization of COVID-19 cases in the early phase (March to July 2020) of the pandemic in Kenya. J Glob Health. 2022;12:15001. doi: 10.7189/jogh.12.15001 36583253 PMC9801068

[12] Minchella PA, Chanda D, Hines JZ, et al. Clinical characteristics and outcomes of patients hospitalized with COVID-19 during the first 4 waves in Zambia. JAMA Netw Open. 2022;5:e2246152. doi: 10.1001/jamanetworkopen.2022.46152 36512359 PMC9856263

[13] Jassat W, Mudara C, Ozougwu L, et al. Difference in mortality among individuals admitted to hospital with COVID-19 during the first and second waves in South Africa: a cohort study. Lancet Glob Health. 2021;9:e1216–e25. doi: 10.1016/S2214-109X(21)00289-8 34252381 PMC8270522

[14] Banda J, Mweemba A, Bulaya T, et al. Predictors of mortality in acute hospitalised COVID-19 pneumonia patients: a retrospective cohort study at two tertiary-level hospitals in Zambia. S Afr Med J. 2022;112:273–278. doi: 10.7196/SAMJ.2022.v112i4.16183 35587806

[15] Russell CD, Lone NI, Baillie JK. Comorbidities, multimorbidity and COVID-19. Nat Med. 2023;29:334–343. doi: 10.1038/s41591-022-02156-9 36797482

[16] van Oostrom SH, Gijsen R, Stirbu I, et al. Time trends in prevalence of chronic diseases and multimorbidity not only due to aging: data from general practices and health surveys. PLOS ONE. 2016;11:e0160264. doi: 10.1371/journal.pone.0160264 27482903 PMC4970764

[17] Nguyen H, Manolova G, Daskalopoulou C, et al. Prevalence of multimorbidity in community settings: a systematic review and meta-analysis of observational studies. J Comorb. 2019;9:2235042X19870934. doi: 10.1177/2235042X19870934 PMC6710708 31489279

[18] Ministry of Health Kenya. Kenya stepwise survey for non communicable diseases risk factors 2015. Nairobi: Ministry of Health; 2015.

[19] World Health Organization. A heavy burden: The productivity cost of illness in Africa. Brazzaville: WHO Regional Office for Africa; 2019.

[20] Achwoka D, Waruru A, Chen TH, et al. Noncommunicable disease burden among HIV patients in care: a national retrospective longitudinal analysis of HIV-treatment outcomes in Kenya, 2003–2013. BMC Public Health. 2019;19:372. doi: 10.1186/s12889-019-6716-2 30943975 PMC6448214

[21] Petersen M, Yiannoutsos CT, Justice A, et al. Observational research on NCDs in HIV-positive populations: conceptual and methodological considerations. J Acquir Immune Defic Syndr. 2014;67:S8–16. doi: 10.1097/QAI.0000000000000252 25117964 PMC4317266

[22] Kharsany AB, Karim QA. HIV infection and AIDS in sub-Saharan Africa: current status, challenges and opportunities. Open AIDS J. 2016;10:34–48. doi: 10.2174/1874613601610010034 27347270 PMC4893541

[23] Smit M, Olney J, Ford NP, et al. The growing burden of noncommunicable disease among persons living with HIV in Zimbabwe. AIDS. 2018;32:773–782. doi: 10.1097/QAD.0000000000001754 29369158 PMC5856639

[24] Mwangi KJ, Mwenda V, Gathecha G, et al. Socio-economic and demographic determinants of non-communicable diseases in Kenya: a secondary analysis of the Kenya stepwise survey. Pan Afr Med J. 2020;37:351. doi: 10.11604/pamj.2020.37.351.24558 33796165 PMC7992900

[25] Western Cape Department of Health, National Institute for Communicable Diseases, South Africa. Risk factors for coronavirus disease 2019 (COVID-19) death in a population cohort study from the Western Cape Province, South Africa. Clin Infect Dis. 2021;73:e2005–e15. doi: 10.1093/cid/ciaa1198 32860699 PMC7499501

[26] Bertagnolio S, Thwin SS, Silva R, et al. Clinical features of, and risk factors for, severe or fatal COVID-19 among people living with HIV admitted to hospital: analysis of data from the WHO Global Clinical Platform of COVID-19. Lancet HIV. 2022;9:e486–e95. doi: 10.1016/S2352-3018(22)00090-1 35561704 PMC9090268

[27] Dzinamarira T, Murewanhema G, Chitungo I, et al. Risk of mortality in HIV-infected COVID-19 patients: a systematic review and meta-analysis. J Infect Public Health. 2022;15:654–661. doi: 10.1016/j.jiph.2022.04.001 35617829 PMC9110010

[28] Etyang AO, Adetifa I, Omore R, et al. SARS-CoV-2 seroprevalence in three Kenyan health and demographic surveillance sites, December 2020-May 2021. PLoS Glob Public Health. 2022;2:e0000883. doi: 10.1371/journal.pgph.0000883 36962821 PMC10021917

[29] National AIDS and STI Control Programme (NASCOP). Preliminary KENPHIA 2018 report. Nairobi: NASCOP; 2020.

[30] Nasimiyu C, Matoke-Muhia D, Rono GK, et al. Imported SARS-CoV-2 variants of concern drove spread of infections across Kenya during the second year of the pandemic. MedRxiv [Prepr]. 2022. doi: 10.1101/2022.04.11.22273676

[31] Ministry of Health Kenya. Interim guidelines on the management of COVID-19 in Kenya. Nairobi: Ministry of Health; 2021.

[32] Onyango EM, Onyango BM. The rise of noncommunicable diseases in Kenya: an examination of the time trends and contribution of the changes in diet and physical inactivity. J Epidemiol Glob Health. 2018;8:1–7. doi: 10.2991/j.jegh.2018.08.004 30859780 PMC7325816

[33] Tessema GA, Kinfu Y, Dachew BA, et al. The COVID-19 pandemic and healthcare systems in Africa: a scoping review of preparedness, impact and response. BMJ Glob Health. 2021;6:e007179. doi: 10.1136/bmjgh-2021-007179 PMC8637314 34853031

[34] Mohamed SF, Haregu TN, Uthman OA, et al. Multimorbidity from chronic conditions among adults in urban slums: the AWI-Gen Nairobi site study findings. Glob Heart. 2021;16:6. doi: 10.5334/gh.891 33598386 PMC7824985

[35] Chudasama YV, Zaccardi F, Gillies CL, et al. Patterns of multimorbidity and risk of severe SARS-CoV-2 infection: an observational study in the UK. BMC Infect Dis. 2021;21:908. doi: 10.1186/s12879-021-06653-2 34481456 PMC8418288

[36] Carmona-Pírez J, Ioakeim-Skoufa I, Gimeno-Miguel A, et al. Multimorbidity profiles and infection severity in COVID-19 population using network analysis in the Andalusian Health population database. Int J Environ Res Public Health. 2022;19:4265. doi: 10.3390/ijerph19074265 35409489 PMC8997853

[37] Hayhoe BW, Powell RA, Barber S, et al. Impact of COVID-19 on individuals with multimorbidity in primary care. Br J Gen Pract. 2022;72:38–39. doi: 10.3399/bjgp22X718229 34972805 PMC8714529

[38] Onyango DO, van der Sande MAB, Musingila P, et al. High HIV prevalence among decedents received by two high-volume mortuaries in Kisumu, western Kenya, 2019. PLOS ONE. 2021;16:e0253516. doi: 10.1371/journal.pone.0253516 34197509 PMC8248726

[39] Yang H, Li X, Stanton B, et al. HIV-related knowledge, stigma, and willingness to disclose: a mediation analysis. AIDS Care. 2006;18:717–724. doi: 10.1080/09540120500303403 16971280 PMC1933389

[40] Ministry of Health Kenya. Kenya HIV estimates report 2022. Nairobi: Ministry of Health; 2023.

