# Supplementary material for: Underlying conditions associated with adverse COVID-19 treatment outcomes in selected Kenyan hospitals, October 2020 to December 2021
Source: Glob Health Action. 2025 Nov 17;18(1):2572010. doi: 10.1080/16549716.2025.2572010 (PMC12624971; doi:10.1080/16549716.2025.2572010)
Supplement: Supplementary _table _clean copy.docx [file ZGHA_A_2572010_SM8330.docx]

**Table S1: Distribution of underlying conditions among patients hospitalized with COVID-19 by waves^§^ of the COVID-19 pandemic, Kenya, Oct 2020 – Dec 2021**

| **Characteristic** | **Total** | **Wave 2** | **Wave 3** | **Wave 4** | **Wave 5** |
| --- | --- | --- | --- | --- | --- |
|  | **N = 1123**  **n (%)** | **N = 128**  **n (%)** | **N = 175**  **n (%)** | **N = 728**  **n (%)** | **N = 88**  **n (%)** |
| **Underlying/Comorbid conditions** |  |  |  |  |  |
| No | 459 (100.0) | 58 (12.6) | 70 (15.3) | 292 (63.6) | 38 (8.3) |
| Yes | 664 (100.0) | 70 (10.5) | 105 (15.8) | 436 (65.7) | 50 (7.5) |
| **Number of Underlying/Comorbid conditions** |  |  |  |  |  |
| 0 | 459 (100.0) | 58 (12.6) | 70 (15.3) | 292 (63.6) | 38 (8.3) |
| 1 | 403 (100.0) | 39 (9.7) | 53 (13.2) | 276 (68.5) | 34 (8.4) |
| 2+ | 261 (100.0) | 31 (11.9) | 52 (19.9) | 160 (61.3) | 16 (6.1) |
| **Hypertension** |  |  |  |  |  |
| No | 714 (100.0) | 83 (11.6) | 103 (14.4) | 454 (63.6) | 73 (10.2) |
| Yes | 409 (100.0) | 45 (11.0) | 72 (17.6) | 274 (67.0) | 15 (3.7) |
| **Diabetes** |  |  |  |  |  |
| No | 865 (100.0) | 98 (11.3) | 126 (14.6) | 561 (64.9) | 77 (8.9) |
| Yes | 258 (100.0) | 30 (11.6) | 49 (19.0) | 167 (64.7) | 11 (4.3) |
| **Cancer** |  |  |  |  |  |
| No | 1117 (100.0) | 124 (11.1) | 175 (15.7) | 727 (65.1) | 87 (7.8) |
| Yes | 6 (100.0) | 4 (66.7) | 0 (0.0) | 1 (16.7) | 1 (16.7) |
| **HIV** |  |  |  |  |  |
| Negative | 11 (100.0) | 2 (18.2) | 1 (9.1) | 7 (63.6) | 1 (9.1) |
| Positive | 94 (100.0) | 9 (9.6) | 11 (11.7) | 60 (63.8) | 13 (13.8) |
| Unknown | 1018 (100.0) | 117 (11.5) | 163 (16.0) | 661 (64.9) | 74 (7.3) |
| **Kidney disease** |  |  |  |  |  |
| No | 1084 (100.0) | 128 (11.8) | 162 (14.9) | 708 (65.3) | 82 (7.6) |
| Yes | 39 (100.0) | 0 (0.0) | 13 (33.3) | 20 (51.3) | 6 (15.4) |
| **Liver disease** |  |  |  |  |  |
| No | 1120 (100.0) | 127 (11.3) | 174 (15.5) | 727 (64.9) | 88 (7.9) |
| Yes | 3 (100.0) | 1 (33.3) | 1 (33.3) | 1 (33.3) | 0 (0.0) |
| **Respiratory diseases** |  |  |  |  |  |
| No | 1053 (100.0) | 118 (11.2) | 165 (15.7) | 687 (65.2) | 80 (7.6) |
| Yes | 70 (100.0) | 10 (14.3) | 10 (14.3) | 41 (58.6) | 8 (11.4) |
| **Heart diseases** |  |  |  |  |  |
| No | 1101 (100.0) | 127 (11.5) | 168 (15.3) | 717 (65.1) | 85 (7.7) |
| Yes | 22 (100.0) | 1 (4.5) | 7 (31.8) | 11 (50.0) | 3 (13.6) |
| **Obesity** |  |  |  |  |  |
| No | 1108 (100.0) | 128 (11.6) | 175 (15.8) | 713 (64.4) | 88 (7.9) |
| Yes | 15 (100.0) | 0 (0.0) | 0 (0.0) | 15 (100.0) | (0.0) |

*Wave was missing for 4 patients which have been exxcluded from this table

**^§^**Wave 2 – October 2020 – December 2020; Wave 3 – February 2021- May 2021; Wave 4 – Jun 2021- October 2021; Wave 5 – November 2021- December 2022
