# Supplementary material for: Underlying conditions associated with adverse COVID-19 treatment outcomes in selected Kenyan hospitals, October 2020 to December 2021
Source: Glob Health Action. 2025 Nov 17;18(1):2572010. doi: 10.1080/16549716.2025.2572010 (PMC12624971; doi:10.1080/16549716.2025.2572010)
Supplement: STROBE_underlying conditions.docx [file ZGHA_A_2572010_SM8329.docx]

STROBE Statement—checklist of items that should be included in reports of observational studies

| \|  \| Item No. \|  \|  \|  \| \| --- \| --- \| --- \| --- \| --- \| \| **Title and abstract** \| 1 \| Underlying conditions associated with adverse COVID-19 treatment outcomes in selected Kenyan hospitals, October 2020 to December 2021 (Page 1) \|  \|  \| \| Abstract (Page 2 \|  \|  \| \| Introduction \| \| \| \|  \| \| Background/rationale \| 2 \| There is limited data on the association between COVID-19 on HIV and non-communicable diseases (Page 3). \|  \|  \| \| Objectives \| 3 \| To examine the distribution of underlying conditions, including NCDs and HIV infection, among people hospitalized with COVID-19 using routine data and explore how these underlying conditions were associated with COVID-19 severity and treatment outcomes. (Page 4) \|  \|  \| \| Methods \| \| \| \|  \| \| Study design \| 4 \| Retrospective cohort study (Page 4) \|  \|  \| \| Setting \| 5 \| Three large COVID-19 treatment centers in Kenya located in Nairobi, Kisumu, and Kilifi Counties (Page 4) \|  \|  \| \| Participants \| 6 \| *Cohort study*— Patients hospitalized for COVID-19 in the participating health facilities between October 1, 2020, and December 31, 2021. Patient were followed up until death or discharge as part of routine care. (Page 4/5) \|  \|  \| \|  \|  \|  \| \| Variables \| 7 \| Outcomes – severe COVID-19 and death; Exposure – HIV, Non-communicable disease, Age, Sex (Page 5) \|  \|  \| \| Data sources/ measurement \| 8* \| We abstracted data from the COVID-19 treatment records onto tablets using a structured form implemented using Open Data Kit (ODK). The extracted variables included socio-demographic characteristics (age in years, sex, and date of hospitalization), clinical characteristics, including underlying conditions, HIV status, and outcome of treatment (death, discharge, or referral). \|  \|  \| \| Bias \| 9 \| Potential selection bias was addressed by including data from three different regions of the country which were known to have large COVID-19 outbreaks. Biases arising from incomplete data were minimized by triangulating multiple data sources \|  \|  \| \| Study size \| 10 \| All medical records for patients hospitalized with COVID-19 in the selected facilities for the  period of interest were included in this study. No sample size was calculated. \|  \|  \|   Continued on next page |  |
| --- | --- | --- | --- | --- | --- | --- | --- | --- | --- | --- | --- | --- | --- | --- | --- | --- | --- | --- | --- | --- | --- | --- | --- | --- | --- | --- | --- | --- | --- | --- | --- | --- | --- | --- | --- | --- | --- | --- | --- | --- | --- | --- | --- | --- | --- | --- | --- | --- | --- | --- | --- | --- | --- | --- | --- | --- | --- | --- | --- | --- | --- | --- | --- | --- | --- | --- | --- | --- | --- | --- | --- | --- |
|  |  |
| \| Quantitative variables \| 11 \| Explain how quantitative variables were handled in the analyses. If applicable, describe which groupings were chosen and why \|  \|  \| \| --- \| --- \| --- \| --- \| --- \| \| Statistical methods \| 12 \| (a) We used chi-square tests to compare the distribution of underlying conditions by the two study outcomes: severity of COVID-19 cases and treatment outcome (alive versus dead) and mixed-effects logistic regression models to account for clustering at facility level and assess the independence of association between underlying conditions and COVID-19 severity and mortality. (Page 5) \|  \|  \| \| (*b*) Describe any methods used to examine subgroups and interactions - None \|  \|  \| \| (*c*) Explain how missing data were addressed -Variables with substantial missing data such as COVID-19 vaccination were excluded from the analysis. (Page 5) \|  \|  \| \| (*d*) *Cohort study*—If applicable, explain how loss to follow-up was addressed – Not applicable \|  \|  \| \| (*e*) Describe any sensitivity analyses _ None \|  \|  \| \| Results \| \| \| \| \| \| Participants \| 13* \| (a) Report numbers of individuals at each stage of study—eg numbers potentially eligible, examined for eligibility, confirmed eligible, included in the study, completing follow-up, and analysed – 1123 individuals included. \|  \|  \| \| (b) Give reasons for non-participation at each stage – Not applicable \|  \|  \| \| (c) Consider use of a flow diagram – Not applicable \|  \|  \| \| Descriptive data \| 14* \| (a) Give characteristics of study participants (eg demographic, clinical, social) and information on exposures and potential confounders – Page 6 \|  \|  \| \| (b) Indicate number of participants with missing data for each variable of interest - Done \|  \|  \| \| (c) *Cohort study*—Summarise follow-up time (eg, average and total amount) – Not applicable \|  \|  \| \| Outcome data \| 15* \| *Cohort study*—Report numbers of outcome events or summary measures over time – Outcomes reported on Page 7. \|  \|  \| \| *Case-control study—*Report numbers in each exposure category, or summary measures of exposure - NA \|  \|  \| \| *Cross-sectional study—*Report numbers of outcome events or summary measures - NA \|  \|  \| \| Main results \| 16 \| (*a*) Give unadjusted estimates and, if applicable, confounder-adjusted estimates and their precision (eg, 95% confidence interval). Make clear which confounders were adjusted for and why they were included - Page 7 \|  \|  \| \| (*b*) Report category boundaries when continuous variables were categorized -NA \|  \|  \| \| (*c*) If relevant, consider translating estimates of relative risk into absolute risk for a meaningful time period - NA \|  \|  \|   Continued on next page |  |
|  |  |
| \| Other analyses \| 17 \| Report other analyses done—eg analyses of subgroups and interactions, and sensitivity analyses \|  \|  \| \| --- \| --- \| --- \| --- \| --- \| \| Discussion \| \| \| \| \| \| Key results \| 18 \| Summarise key results with reference to study objectives – Page 7 \|  \|  \| \| Limitations \| 19 \| Discuss limitations of the study, taking into account sources of potential bias or imprecision. Discuss both direction and magnitude of any potential bias – Page 8 \|  \|  \| \| Interpretation \| 20 \| Give a cautious overall interpretation of results considering objectives, limitations, multiplicity of analyses, results from similar studies, and other relevant evidence – Pages 7/8 \|  \|  \| \| Generalisability \| 21 \| Discuss the generalisability (external validity) of the study results – Page 7/8 \|  \|  \| \| Other information \| \|  \| \| \| \| Funding \| 22 \| Give the source of funding and the role of the funders for the present study and, if applicable, for the original study on which the present article is based – Page 9 \|  \|  \| |  |

*Give information separately for cases and controls in case-control studies and, if applicable, for exposed and unexposed groups in cohort and cross-sectional studies.

**Note:** An Explanation and Elaboration article discusses each checklist item and gives methodological background and published examples of transparent reporting. The STROBE checklist is best used in conjunction with this article (freely available on the Web sites of PLoS Medicine at http://www.plosmedicine.org/, Annals of Internal Medicine at http://www.annals.org/, and Epidemiology at http://www.epidem.com/). Information on the STROBE Initiative is available at www.strobe-statement.org.
